# Effect of Diet Compositions on Colony Strength Parameters, and the Enzymatic Activity of *Apis mellifera* L. During Floral Scarcity

**DOI:** 10.3390/insects16090967

**Published:** 2025-09-16

**Authors:** Shams Ul Islam, Javeria Liaquat, Muhammad Anjum Aqueel, Asim Abbasi, Muhammad Arshad, Muhammad Shahid Rizwan, Muhammad Saqib, Nasir Masood, Nyasha J. Kavhiza, Saba Zafar, Graciela Dolores Avila-Quezada, Elsayed Fathi Abd_Allah, Dalal Saad Alharbi, Abeer Hashem

**Affiliations:** 1 Department of Entomology, Faculty of Agriculture and Environment, The Islamia University of Bahawalpur, Bahawalpur 63100, Pakistan; 2 Department of Entomology, University of Agriculture, Faisalabad 38040, Pakistan; asimuaf5@gmail.com (A.A.);; 3 Cholistan Institute of Desert Studies, Faculty of Agriculture & Environment, The Islamia University of Bahawalpur, Bahawalpur 63100, Pakistan; 4 Department of Agronomy, Faculty of Agriculture and Environment, The Islamia University of Bahawalpur, Bahawalpur 63100, Pakistan; 5 Department of Biosciences, Chak Shahzad, Tarlai Kalan, COMSATS University Islamabad, Islamabad 45550, Pakistan; 6 Department of Environmental Management, Institute of Environmental Engineering, RUDN University, 6 Miklukho-Maklaya St., 117198 Moscow, Russia; 7 Department of Biochemistry and Biotechnology, The Women University Multan, Multan 66000, Pakistan; 8 Facultad de Ciencias Agrotecnológicas, Universidad Autónoma de Chihuahua, Chihuahua 31350, Chihuahua, Mexico; 9 Department of Plant Production, College of Food Science and Agriculture, King Saud University, P.O. Box 2460, Riyadh 11451, Saudi Arabia; 10 Department of Botany and Microbiology, College of Science, King Saud University, P.O. Box 2455, Riyadh 11451, Saudi Arabia

**Keywords:** artificial diet, honey bee nutrition, *Apis mellifera* L., colony performance, dearth period, enzymatic activity

## Abstract

Dearth periods associated with less floral resources negatively impact *Apis mellifera* colony performance. Artificial diets offer nutritional supplements to sustain bee colonies under stressful conditions. An eight-week feeding trial was conducted using various artificial diets (eight diets, including a control diet), formulated with varying quantities of pulses, yeast, fenugreek powder, vegetable oil, dry apricot powder, and powdered sugar. Colony performance of bees subjected to different artificial diets was evaluated based on diet consumption, brood area, adult bee population, worker bee longevity, honey production, and enzymatic activity. Diet-7, which uniquely combined lupin, mung bean, and chickpea flours, proved the most efficacious and was the most consumed diet (84.29 ± 1.61 g), while diet-1 showed the lowest consumption (35.30 ± 1.08 g). Maximum brood area was recorded in colonies which were offered diet-7 (1385.95 ± 14.91 cm^2^), followed by diet-6, whereas the lowest was observed in the control (831.03 ± 18.95 cm^2^). The adult bee population was highest in diet-7 (21,594.50 ± 94.55 bees/hive), while lowest in the control (diet-0) (12,625.43 ± 385.06 bees/hive). Worker bee longevity was greatest in diet-7 (49.40 ± 0.41 days) and lowest in the control group (37.01 ± 0.39 days). Honey production was also highest in diet-7 (8.86 ± 0.21 kg), while lowest in the control (2.79 ± 0.35 kg). The results further showed that the enzymatic activities of bees were significantly improved due to diet-7, with the highest values for amylase (48.62 ± 0.23 U/mg), lipase (16.85 ± 0.20 U/mg), proteinase (25.21 ± 0.18 U/mg), and α-glucosidase (39.21 ± 0.21 U/mg). In conclusion, statistical analyses confirmed that diet-7 emerged as the most effective artificial diet, enhancing colony performance across all evaluated parameters. Future research should aim to optimize diet formulations and evaluate their effectiveness on colony health, including gut microbiome and immune function, across different seasons and ecological regions.

## Simple Summary

Artificial diets play an important role in fulfilling the nutritional requirement of bee colonies especially during dearth periods. In the current study, honey bee colony performance subjected to different artificial diets was evaluated based on diet consumption, brood area, adult bee population, worker bee longevity, honey production, and enzymatic activity. The results showed that bees preferred diet-7 (20 g lupin flour + 20 g mung bean flour + 20 g chickpea flour + 2 g fenugreek powder + 10 g yeast + 40 g powder sugar + 10 g dry apricot powder + 10 mL vegetable oil), which also significantly improved brood area, bee population, worker bee longevity, honey output, and enzyme activity of worker bees, highlighting the significance of targeted nutrition under floral scarcity. These findings underscore the pivotal role of targeted nutritional supplementation in maintaining optimal physiological functioning and social organization within honey bee colonies, particularly during nutritional stress. However, future research should prioritize the optimization of artificial diets using low-cost, locally available, palatable, and shelf-stable ingredients to support local honey bee populations during floral scarcity.

## 1. Introduction

Animal pollination, particularly by insects, is a vital ecosystem service that enhances fruit setting and yield in both staple and cash crops [1,2,3]. Animal pollination, particularly through insects, contributes to nearly one-third of the human diet globally. Although pollinators comprise a wide spectrum of insects, researchers seem to focus mainly on honey bees due to their significant role in crop pollination [4]. Furthermore, among different honey bees, *Apis mellifera* L. provides highly valued pollination services for a wide range of crops [4], and is recognized as the most common pollinator for numerous crops worldwide [5]. In addition to their pollination services, honey bees including *A. mellifera* provide products such as honey, wax, propolis, beebread, and royal jelly, all of which offer significant benefits to humans. However, despite the significant importance of *A. mellifera* in agriculture, its role as a key pollinator in natural ecosystems remains less studied [6,7].

During certain times of the year such as hot summers, cold winters, and rainy seasons, the availability of floral resources (nectar and pollens) makes colony survival challenging [8]. These dearth periods demand the development and provision of artificial diets to compensate for the limitations of natural bee flora [9]. Colonies without access to natural pollen, which is the primary protein source for the bees, are unable to rear sufficient brood [10], leading to rapid population decline and possible colony collapse [10]. Worldwide, various artificial diets have been formulated based on bees’ nutritional needs, aiming to sustain colony health [11]. Scientists feed artificial diets to bee colonies and assess their effects on nutritional quality and colony performance [12]. Sugar syrup supplementation is also commonly practiced by the majority of beekeepers to enhance brood development and oviposition [13]. However, only sugar syrup is not sufficient to feed on as a diet for honey bees to survive better [14] and for their optimal growth [15]. Malnutrition exerts detrimental impacts on honey bee colonies by compromising their health and overall performance [16], while simultaneously increasing their susceptibility to insect pests [17], disease outbreaks [18], and colony collapse disorder [19]. Therefore, it is essential to provide pollen-based alternative diets to bee colonies for their subsistence and development [20]. The effectiveness of these diets can be calculated usually through diet consumption, brood area measurements [21,22,23], worker bee longevity, and other colony performance indicators [24].

The composition of different honey bee diets also impacts their enzymatic activity [25]. It usually begins when bees collect nectar and continues through regurgitation and hive storage [26]. Amylase, secreted by the bee salivary glands, is a key enzyme used in the conversion of nectar into honey. It also breaks down complex carbohydrates into simpler sugars such as glucose and maltose [27], thereby aiding in sweetening, stabilizing, and extending the shelf life of honey [28]. Lipase, another enzyme found in many insect species, hydrolyzes triglycerides into glycerol and free fatty acids [29,30]. It plays a vital role in energy metabolism during periods of starvation by mobilizing fat reservoirs [31]. Proteinases are essential for physiology and development in honey bees. They break down peptide bonds, producing smaller peptides and amino acids [32]. Proteinase activity is crucial for digesting pollen, which is the principal source of protein for honeybees [33], and supports adult health, larval growth and development, and royal jelly production [34]. Additionally, it degrades pathogen defense proteins, aiding immune regulation and overall colony health maintenance [35]. Another enzyme present in the hypopharyngeal gland of honeybees is called α-glucosidase, which hydrolyzes α-(1,4) bonds into monosaccharide units [36]. The α-glucosidase is liberated in bee saliva and helps in the enzymatic alteration and regurgitation of nectar [37], enhancing the flavor, durability, and antimicrobial properties of honey, while reducing its crystallization potential [38]. The conversion of nectar into honey is a highly regulated and nutritive process, which requires the crucial step of invertase activity [39].

Keeping in view the above facts, the present study was designed to evaluate the effectiveness of various artificial diets in supporting the survival and physiological performance of *A. mellifera* colonies during the dearth period, with particular emphasis on enzymatic activity, colony development, honey production, and worker bee longevity. This study hypothesizes that supplementation with nutritionally enriched artificial diets would improve these parameters, thereby enhancing overall colony health. However, future studies must consider the performance of promising artificial diets across diverse ecological regions and climatic conditions to validate their broader applicability. Moreover, exploring their impact on immune function and disease resistance using molecular and biochemical methods may provide deeper insights into how artificial nutrition modulates enzymatic activity and essential metabolic processes in honey bees.

## 2. Materials and Methods

### 2.1. Apiary Setup and Experiment Layout

This study was conducted in the Apiculture Research Area at the Department of Entomology, The Islamia University of Bahawalpur (29°22′25″ N or 71°45′53″ E), Bahawalpur, Punjab, Pakistan. This region experiences hot summers with temperatures ranging from almost 40–45 °C and mild winters with temperatures ranging from almost 9–23 °C. The harsh weather during intense summers usually results in fewer floral resources, making it difficult for pollinators to survive. However, during the winter months, beekeepers from the colder northern regions of Pakistan migrate to Bahawalpur to benefit from the abundant nectar and pollen available in the mustard fields. The experimental procedure involved twenty-four healthy and disease-free colonies having a fertile queen. Each colony initially had nine frames and ~15,000 bees. The colonies were randomly distributed among eight treatments, including the control, with each treatment replicated three times. Experiments were conducted from 1 June 2024 to 30 August 2024.

### 2.2. Diet Preparation

Diet ingredients were selected based on their nutrient contents (such as protein, vitamins, carbohydrates, fats, and minerals), variation in texture and flavor, cost-effectiveness, availability, and cited literature. The different diet ingredients used in the current study included chickpea, lupin, and mung bean (a source of protein) [40], fenugreek powder (a source of fiber, vitamin B, C, K, and minerals) [41], dry apricot powder (source of vitamins, minerals, iron) [42], bakery yeast as a source of protein, vitamin B, and minerals [43], powdered sugar as a source of carbohydrate [44], and vegetable oil as a source of fats [45]. These diet ingredients were used in different combinations. A systematized sugar solution was prepared with a ratio of 1:1 (50% concentration) by liquefying pure sucrose in clean water for the control treatments.

The lentils were ground separately in a grinding machine and later mixed with the rest of the ingredients. The specified amount of each lentil flour was added to a bowl amended with dry apricot powder, bakery yeast, powdered sugar, and vegetable oil. The mixture was blended thoroughly in a dough mixer. The detailed composition of different diets is given in Table 1.

### 2.3. Diet Consumption

Prepared diets were filled in Petri dishes in equal amounts (100 g) and subsequently wrapped with a polythene sheet (to maintain moisture levels), leaving the space for honeybees to eat the diets [46]. The diet patties were checked after 24 h and replaced with fresh diets after every seven days [47]. For the control group (diet-0), 1 L of 50% sugar solution was provided at the start of each week in bee feeders placed at the position of the 10th frame. Data was collected over a period of 8 weeks, during which the quantity of food consumed was calculated by noting the difference between the weight of the fresh diet patty and the weight of the patty after a week [48].

### 2.4. Brood Area Measurement

The brood area was calculated from all the frames of each replication of experimental treatments. A modified grid arrangement was used to measure the brood area. The square of the grid has a surface area of 25 cm^2^ [49]. The grid was used to measure and mark the sealed brood frame area [50]. Brood frames were identified by visually examining both sides of the comb; open brood (eggs and unsealed larvae) and sealed brood (capped cells) were recorded separately using a transparent grid. By arranging the grids and brood frames in a specific way, a pattern was created that helps in visualizing the entire area covered by the brood. The number of empty cells was subtracted, and then the average was taken [51,52]. The calculation was performed by using a standardized formula: the number of brood frames was multiplied by the corresponding percentage of brood coverage on each frame, as given in previous research [53].

### 2.5. Adult Bee Population

After feeding artificial diet to bee colonies, the adult bee population was calculated following the methods described by Delaplane et al. [54]. This process involved a complete count of frames (both sides of the frame) to assess the population and strength of the colony [55]. A sample of 300 bees was collected from the frames and weighed to establish a reference scale for population estimation. The weight of 300 bees used as the reference scale was 29.1 g (29.1 g/300 bees). All frames from each hive, across treatment groups and replications, were weighed both with bees present and again after gently brushing off the bees. The difference in weight was used to estimate the adult bee population, based on the previously established scale [54].

### 2.6. Worker Bee Longevity

As the adult bees emerged from the brood cells, they were marked using a permanent non-toxic color marker (POSCA PC-5M, Mitsubishi Pencil Co., Ltd., Boulogne-Billancourt Cedex, France), following the method described by Manzoor et al. [47] and Shurjeel et al. [56]. From each colony, 15 newly emerged worker bees were marked. The date and time of emergence were recorded for each marked bee. Bee longevity was assessed by daily inspecting each colony at 10:00 am, identifying the presence or absence of the marked bees. Observations continued until all marked bees were no longer found in the hive, indicating their death or disappearance [47].

### 2.7. Honey Production

In the end, honey production was also computed in order to compare the output of bee colonies. The honey extractor machine was used to extract honey from all the colonies, making sure all the standard procedures of honey extraction were taken into account [57]. Statistically analyzed production data was used to examine results [58].

### 2.8. Enzymatic Analysis

After eight weeks of diet consumption, 30 worker honey bee samples were collected from each experimental treatment group (10 from each replication) [24], for enzymatic analysis. The gut was extracted from all the bee samples, homogenized in chilled tris-HCl (50 mm), and taken in centrifuge tubes. Then the samples were centrifuged (6000 rpm) at 4 °C for 15 min [59]. The supernatant was collected and stored at 0 °C. Three replicates were used for each sample.

#### 2.8.1. Amylase Assay

A starch substrate was used to determine amylase activity [60]. A sample of 1 mL, which was diluted and mixed with 1% starch substrate (1 mL), was incubated for 3 min at a temperature of 37 °C. Reagent 3.5-dinitro salicylic acid (2 mL) was added to this mixture and monitored until the reaction stopped [61]. The solution was placed in boiling water and heated for 5 min, after which it was cooled down, and amended with 20 mL of distilled water (20 mL). Absorbance was checked against the blank at the 540 nm wavelength [59].

$$\text{Enzyme activity}\left(\frac{U}{\mathrm{mL}}\right)=\frac{\Delta \text{A enzyme}-\Delta \text{A blank}}{\left(\text{incubation time}\times \text{dilution factor}\right)\times \text{standard factor}}$$

#### 2.8.2. Lipase Assay

In addition, 1 mL of the sample, the buffer phosphate (0.2 M, pH 6.9), and olive oil with a quantity of 0.5 mL were added to a glass flask with a capacity of 3.5 mL. Following that, the mixture was agitated for 30 min at a temperature of 37 °C in a water bath. Then, acetic acid in the quantity of 1 mL was added to the mixture, and it was titrated with base NaOH (10 mm) until the pH of the solution achieves a value of 10 [59]. The lipase activity was measured using the following formula:

$$\text{Enzyme activity}\left(\frac{U}{\mathrm{mL}}\right)=\frac{\left(\text{NaOH vol.}\times \text{Molarity of NaOH}\times 1000\times 2\times \mathrm{df}\right)}{\left(\mathrm{volume}\right)}$$

#### 2.8.3. Proteinase Assay

Tyrosine standard solutions (0.1 to 0.5 μmol/mL) were prepared along with a blank. For each standard, 2 mM of the tyrosine solution was transferred into a series of test tubes, followed by the addition of 5.0 mL Na_2_CO_3_ and 1.0 mL Folin–Ciocalteu phenol reagent. The mixture was stirred, incubated at 37 °C for 30 min, and then filtered through a 0.45 μm syringe filter. Absorbance was measured at 660 nm, and blank values were subtracted. For proteinase activity determination, 5 mM casein solution was mixed with 1.0 mL enzyme extract in a test tube and incubated at 37 °C for 5 min. The enzymatic reaction was stopped by adding 5.0 mL trichloroacetic acid reagent, followed by incubation at 37 °C for 30 min. The mixture was filtered through a 0.45 μm syringe filter, and 2 mM of each filtrate was combined with 5.0 mL Na_2_CO_3_ and 1 mL 0.5 M Folin–Ciocalteu phenol reagent. After thorough mixing, the samples were incubated at 37 °C, and absorbance was recorded at 660 nm, with blank corrections applied [62,63].

#### 2.8.4. α-Glucosidase Assay

Extracts at varying concentrations (0.01 to 200 g/mL) were preincubated with α-Glucosidase (0.075 unit). The reaction was initiated by adding p-nitrophenyl-D-glucopyranoside (3 mM) as the substrate to the phosphate buffer contained in the reaction tube. The mixture was incubated at 37 °C for 30 min, after which the reaction was terminated by the addition of Na2CO3. The activity of the α-glucosidase was determined by measuring the release of p-nitrophenol from p-nitrophenyl-D-glucopyranoside at 400 nm and expressed in U/mL [64].

### 2.9. Data Analysis

All statistical analyses were performed using R software (R Core Team, Vienna, Austria, 2024 version 4.4.2). To evaluate the effects of different artificial diets on the studied parameters, a two-way factorial design was employed for diet consumption data, considering diet type and feeding week as two treatment factors. This design allowed the assessment of both main effects and their interaction. A two-way Analysis of Variance (ANOVA) was used to test the statistical significance of the main and the interaction effects. Prior to analysis, data were checked for normality (Shapiro–Wilk test) and homogeneity of variances (Levene’s test). Since the assumptions were met, no data transformation was required. Where significant effects were observed, the Least Significant Difference (LSD) test at the *p* < 0.05 level was applied to differentiate treatment means. In addition, for other parameters (e.g., colony strength indicators), one-way ANOVA was conducted where applicable. Where significant differences were observed, the LSD test was similarly used for post hoc comparisons. For field trials, a significance level of *p* < 0.05 was used, while a more stringent threshold of *p* < 0.01 was applied in laboratory trials to ensure higher precision under controlled conditions. Graphical representations of the data, including mean values with standard errors of the mean (SE), were constructed using the ggplot2 (version 3.5.1) and dplyr (version 1.1.4) packages in the same software.

## 3. Results

### 3.1. Diet Consumption

All treatments (diets), time intervals (weeks), and their interactions were analyzed for their effects on diet consumption. The analysis of variance revealed that both weeks (F(7,110) = 31.14, *p* < 0.05) and treatments (F(6,110) = 708.43, *p* < 0.05) had highly significant effects on diet consumption, whereas the interaction between weeks and treatments (F(42,110) = 0.27, *p* = 1.00) was not significant (Table 2).

During week 1, a statistically significant difference was observed among the seven diets (*p* < 0.05) regarding their consumption. The highest consumption was recorded for diet-7 (79.90 ± 2.19 g), followed by diet-6 (65.50 ± 2.10 g) and diet-5 (58.22 ± 1.45 g), while the lowest consumed diet was diet-1 (37.42 ± 1.60 g). In week 2, diet-7 again showed the highest consumption (78.37 ± 2.01 g), followed by diet-6 and diet-5, with diet-1 being the least consumed. The same trend had been observed during week 3, in which the highest consumption was recorded for diet-7 (77.58 ± 1.74 g) and the least for diet-1 (35.30 ± 1.08 g) (Table 2).

In week 4, bees again preferred diet-7 with maximum consumption of 76.04 ± 1.94 g; however, the minimum consumed diet was diet-1 (36.41 ± 0.94 g). Similar trends were recorded for the rest of the week intervals. Week 5 showed the highest intake from diet-7 (83.41 ± 2.07 g), followed by diet-6 (70.12 ± 0.92 g) and diet-5 (62.51 ± 2.30 g), with the least intake from diet-1 (43.86 ± 1.92 g). In week 6, diet-7 remained the most consumed (78.73 ± 1.48 g) and diet-1 as the least consumed (39.97 ± 1.30 g). During week 7, consumption peaked again for diet-7 (84.29 ± 1.61 g), followed by diet-6 and diet-5. In the final week (week 8), diet-7 showed the highest intake (83.30 ± 2.27 g), while diet-1 exhibited the lowest intake (43.29 ± 1.64 g). Overall, the results showed that bees preferred diet-7 (highest consumption) during the whole course of the study, followed by diet-6 and diet-5 (Table 2).

### 3.2. Brood Area Measurement

**Figure 1:**
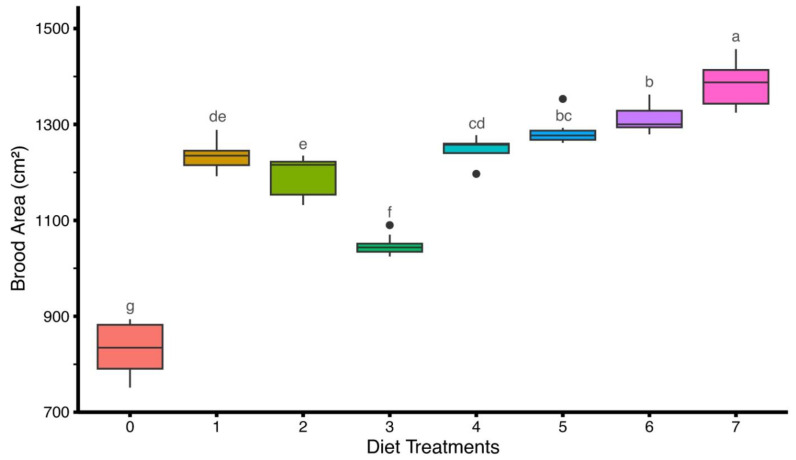
Effect of different artificial diets on brood area (cm^2^) of *Apis mellifera*. Values represent the mean brood area per diet ± standard error (SE). Different lowercase letters (a, b, c, etc.) indicate significant differences among treatments at 5% significance level (*p* < 0.05).

A significant difference (F(7,64) = 207, *p* < 0.05) was observed in the brood area (cm^2^) of honey bee colonies fed on different artificial diets. The results showed that the highest brood area was recorded in diet-7 (1385.95 ± 14.91 cm^2^), followed by diet-6 (1312.80 ± 9.73 cm^2^), diet-5 (1284.55 ± 9.17 cm^2^), and diet-4 (1245.44 ± 9.80 cm^2^). Moderate brood areas were noted in diet-1 (1236.13 ± 10.74 cm^2^) and diet-2 (1191.07 ± 13.31 cm^2^). Excluding the control group (diet-0: 831.03 ± 18.95 cm^2^), diet-3 exhibited the smallest brood area (1048.75 ± 6.79 cm^2^) among the tested artificial diets (Figure 1).

### 3.3. Adult Bee Population

**Figure 2:**
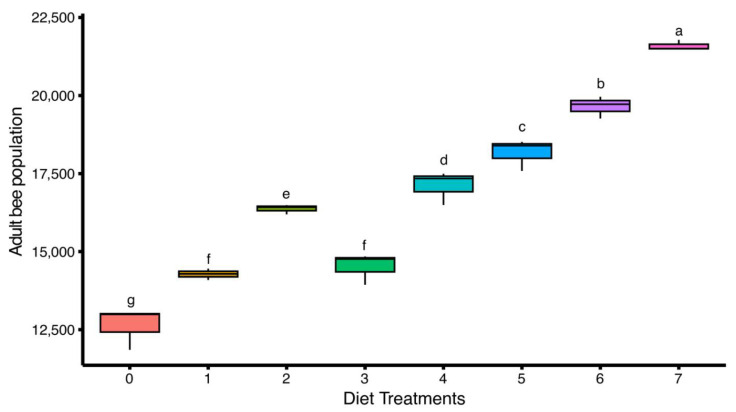
Effect of different artificial diets on adult bee population of *Apis mellifera*. Values represent the mean population per treatment group (Diet) ± standard error (SE). Different lowercase letters (a, b, c, etc.) indicate significant differences among treatments at 5% significance level (*p* < 0.05).

A significant difference (F(7,16) = 148.00, *p* < 0.05) in the adult bee population was observed among colonies fed with different artificial diets. The highest adult bee population was recorded in diet-7 (21,594.50 ± 94.55 bees/hive), followed by diet-6 (19,649.48 ± 202.63 bees/hive) and diet-5 (18,164.95 ± 290.86 bees/hive), indicating a strong positive effect of these diets on colony strength. Moderate populations were found in colonies fed with diet-4 (17,109.97 ± 310.78 bees/hive) and diet-2 (16,367.70 ± 87.75 bees/hive). Diet-3 and diet-1 showed relatively lower populations, measuring 14,515.46 ± 289.64 bees/hive and 14,278.35 ± 104.29 bees/hive, respectively. The lowest adult bee population was observed in diet-0 (control), with a mean value of 12,625.43 ± 385.06 bees/hive (Figure 2).

### 3.4. Life Span of Worker Bees

**Figure 3:**
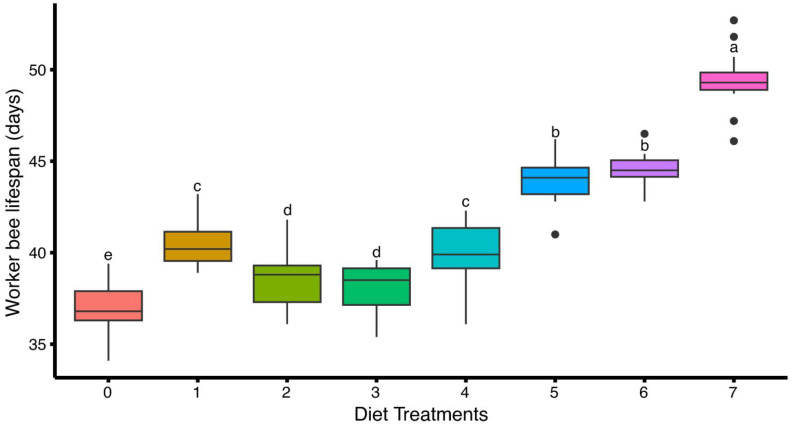
Effect of different artificial diets on worker bee lifespan of *Apis mellifera*. Values represent the mean longevity per treatment group (Diet) ± standard error (SE). Different lowercase letters (a, b, c, etc.) indicate significant differences among treatments at 5% significance level (*p* < 0.05).

A significant difference in worker bee longevity was observed among bees fed with different artificial diets (F(7,112) = 134.00, *p* < 0.05). The highest worker bee longevity was recorded in diet-7 (49.40 ± 0.41 days), followed by diet-6 (44.53 ± 0.25 days) and diet-5 (43.97 ± 0.32 days), indicating a strong positive effect of these diets on extending worker lifespan. Moderate longevity was observed in bees fed with diet-1 (40.44 ± 0.30 days) and diet-4 (40.05 ± 0.45 days), while relatively lower values were recorded in the case of diet-2 (38.59 ± 0.39 days) and diet-3 (38.04 ± 0.33 days). The lowest worker bee longevity was observed in diet-0 (control), with a mean of 37.01 ± 0.39 days (Figure 3).

### 3.5. Honey Production

**Figure 4:**
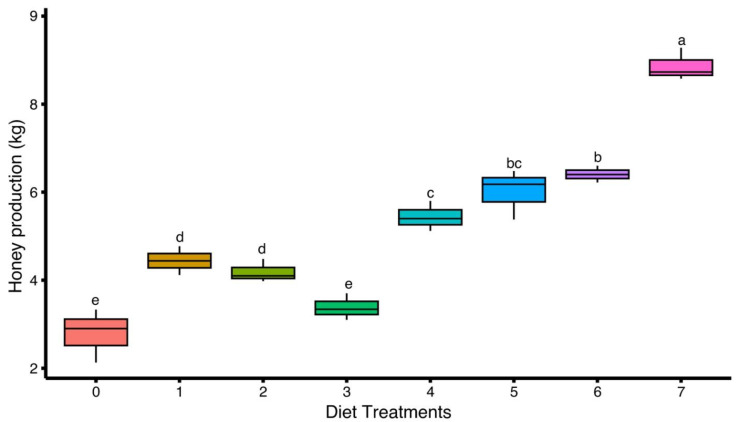
Effect of different artificial diets on honey production of *Apis mellifera*. Values represent the mean honey yield per treatment group (Diet) ± standard error (SE). Different lowercase letters (a, b, c, etc.) indicate significant differences among treatments at 5% significance level (*p* < 0.05).

A significant difference in honey production was observed among colonies fed with different artificial diets (F(7,16) = 72.4, *p* < 0.05), indicating the strong effect of diet composition on honey production. The highest honey yield was recorded in diet-7 (8.86 ± 0.21 kg), followed by diet-6 (6.41 ± 0.11 kg) and diet-5 (6.01 ± 0.33 kg), highlighting the positive impact of these diets on nectar foraging and conversion efficiency. A moderate level of production was observed in diet-4 (5.44 ± 0.20 kg), while diet-1 (4.44 ± 0.19 kg) and diet-2 (4.19 ± 0.15 kg) showed comparatively lower yields. However, the lowest honey production was recorded in the control group, diet-0 (2.79 ± 0.35 kg) (Figure 4).

### 3.6. Pearson’s Correlation Matrix

A strong correlation was observed between brood area and adult bee population (r = 0.856), indicating that colonies with larger brood areas tend to support more adult bees. Similarly, brood area and honey production were positively associated (r = 0.856), suggesting that increased brood rearing capability enhances foraging output and productivity. The strongest correlation was found between adult bee population and honey production (r = 0.957), underscoring the critical role of colony strength in maximizing nectar foraging and honey storage. Worker bee longevity also showed higher positive correlations with brood area (r = 0.804), adult bee population (r = 0.920), and honey production (r = 0.975), suggesting longer bee lifespans also support colony development and productivity. The healthier, stronger colonies (larger brood and population) inherently produce more honey and have workers with extended lifespans (Table 3).

### 3.7. Enzymatic Analysis

Significant variations (*p* < 0.01) were observed in the activities of amylase, lipase, proteinase, and α-glucosidase enzymes among the different dietary treatments. The results revealed that the highest amylase activity was recorded in diet-7 (48.62 ± 0.23 U/mg), which was statistically at par with diet-5 (47.50 ± 0.29 U/mg) and diet-6 (47.00 ± 0.52 U/mg), and significantly higher than diet-0 (40.66 ± 0.61 U/mg) (F(7,16) = 43.2, *p* < 0.01). Moderate activity was observed in diet-1 (46.09 ± 0.46 U/mg) and diet-4 (46.11 ± 0.35 U/mg), while lower values were found in diet-2 (45.28 ± 0.43 U/mg) and diet-3 (42.01 ± 0.29 U/mg). Lipase activity followed a similar trend, with diet-7 (16.85 ± 0.20 U/mg) showing the highest value, significantly surpassing all other treatments (F(7,16) = 52.5, *p* < 0.01). Similarly, diet-6 (16.25 ± 0.14 U/mg) and diet-5 (16.00 ± 0.14 U/mg) also exhibited elevated activity, while the lowest was observed in the control group diet-0 (11.93 ± 0.35 U/mg). Proteinase activity peaked in diet-7 (25.21 ± 0.18 U/mg), followed by diet-5 (24.11 ± 0.12 U/mg) and diet-6 (23.10 ± 0.17 U/mg) (F(7,16) = 171, *p* < 0.01). Diet-0 (17.60 ± 0.26 U/mg) showed the lowest activity, significantly different from all supplemented diets. The activity of α-glucosidase was also significantly affected (F(7,16) = 333, *p* < 0.01), with diet-7 (39.21 ± 0.21 U/mg) recording the highest value. It was statistically similar to diet-5 (38.76 ± 0.15 U/mg) but significantly greater than diet-0 (29.26 ± 0.21 U/mg). Diet-1 and diet-2 also showed intermediate levels of activity (Table 4).

## 4. Discussion

The study emphasized how different artificial diets, made from various protein-rich items like pulses, yeast, vegetable oils, sugar syrup, and dry apricot powder, affected the key performance parameters of *Apis mellifera* colonies over an eight-week period. Diet consumption, brood area, adult bee population, enzymatic activity, and worker bee lifespan were evaluated in response to different feeding diets.

The variation in diet consumption among treatments underscores the importance of palatability and digestibility in diet design. A diet’s acceptance is the initial determinant of its effectiveness, as bees will not consume food that lacks desirable organoleptic properties. The present results align with those of Manzoor et al. [47], who used similar cost-effective ingredients to formulate bee diets. This observation also supports the findings of Taha and Al-Kahtani [65], who emphasized that sugar-protein balance significantly influences dietary uptake, and Abou-Shaara [3], who reported that texture and odor can impact diet attractiveness. Both studies concluded that poor acceptability compromises overall colony nutrition, a phenomenon not observed in the more accepted diets in the present study. Our findings further suggest that the balance of macronutrients (proteins, lipids, carbohydrates) and micronutrients (minerals, vitamins, phytochemicals) is critical for brood rearing. Previous studies have reported that excess protein without complementary lipids and sterols may impair digestion and colony health [66,67]. Therefore, the better efficacy of particular diets used in the present study might be associated with the presence of not only higher protein contents but also a balanced ratio of other important nutrients.

Brood area measurement is directly relevant to colony health. Nurse bees require sufficient protein to produce royal jelly and nourish larvae; therefore, protein-enriched diets for honey bees have consistently resulted in increased brood numbers. Noordyke and Ellis [68] discovered that soy and yeast in the diet improved brood production more than a sucrose-only diet. Similarly, DeGrandi-Hoffman et al. [69] observed that using pollen substitutes in addition to natural pollen increased both larval development and the number of capped brood cells. According to recent findings, balanced artificial foods enable bees to continue producing young ones even during dearth periods. Worker bee lifespan was determined by daily monitoring of marked bees, recording only natural in-hive deaths; bee drift or absconding did not affect the data.

A healthy adult bee population depends on both the colony’s vitality and the success of brood emergence, both of which are influenced by larval feeding and the nutritional state of the colony. Colonies fed specially designed diets had a larger number of offspring and lower adult mortality, indicating improved outcomes for both broods and adults. These results align with Nabors [70], who demonstrated that protein-rich diets increase bee numbers and colony development, and Mattila and Otis [71], who found that both the amount and quality of food consumed by workers are crucial for population growth and colony strength. The sustained presence of adult bees in this study can thus be attributed to the continuous supply of essential nutrients in the artificial diets provided. Similarly, previous studies have reported that plant-derived supplements such as fenugreek powder and legume powders significantly enhanced the efficiency, survival, and overall health of honey bees [72,73].

A colony’s ability to forage, regulate temperature, and feed its young ones relies heavily on worker bee longevity. Longer lifespans reduce physiological stress and enhance immunity, both of which are strongly influenced by diet. Standifer et al. [74] were the first to prove the importance of protein for bees in laboratory trials, while Saffari et al. [75] demonstrated that bees fed protein during pollen shortage lived significantly longer. The current findings confirm that proper nutrition helps colonies maintain younger populations and coordinate work more effectively during food scarcity.

The convergence of high diet acceptability, brood development, enzymatic activity, and longevity under specific treatments underscores the multidimensional impact of artificial nutrition. Previous research underlines that both nutritional quality and digestibility should be considered when formulating artificial diets for honey bees [65,69]. If diets fail to meet these criteria, their effectiveness in sustaining colonies can be inconsistent or limited, as reported by Pankiw et al. [76] and also confirmed in the present study.

Honey production depends on foraging ability, colony size, and overall metabolism, all of which are influenced by diet during the dearth period. Colonies fed nutritious artificial diets in this study produced more honey, suggesting extended worker lifespans, improved brood care, and enhanced foraging efficiency. These results are in agreement with Abou-Shaara [77], who noted that artificial diets promote colony development and encourage nectar collection and honey storage. Similarly, El-Kazafy and Ali [78] found that providing diets rich in protein and energy significantly increased honey production in *A. mellifera* colonies, particularly when floral resources were scarce. This research further supports the role of proper nutrition in maintaining honey bee health and boosting productivity.

During food shortages, the activity of key enzymes in a honey bee colony such as proteases serves as an indicator of nutritional status and colony health. Protein-rich diets enhance the bee’s ability to digest protein, which is essential for brood rearing and gland development [79,80]. Similarly, a carbohydrate-rich diet increases amylase activity, vital for energy-demanding processes such as foraging and thermoregulation [81]. Elevated invertase activity supports honey processing and immediate energy demand [82], while higher glucose oxidase activity enhances the bees’ natural immune defense by producing antiseptic substances that protect the colony from diseases [83]. Collectively, these enzymatic responses underscore the nutritional benefits of the tested diets and their role in enhancing colony resilience and survival during nutritionally stressful periods. Collectively, these enzymatic responses indicate enhanced digestion and utilization of nutrients from the artificial diets. Recent studies also emphasize that diet composition directly affects the honey bee gut microbiome and gene expression. Pollen-free artificial diets often reduce microbial diversity and beneficial bacteria [84], whereas inclusion of pollen increases gut weight and bacterial loads [85]. Such findings suggest that protein-rich ingredients in artificial diets (e.g., pulses, yeast) may help sustain a healthy microbiome during dearth periods, supporting bee health and colony resilience.

Beyond dearth periods, the diet of overwintering “diutinus” worker bees differs markedly from that of summer foragers. During late autumn and winter, these bees cluster together to maintain colony warmth and subsist exclusively on stored provisions, including pollen, bee bread, and honey (rather than fresh forage) [71,86]. This dietary shift drives notable changes in gut microbiota, including higher bacterial loads, greater *Lactobacillus* abundance, and increased *Bartonella* spp. [87]. Such changes underscore the critical role of winter diet in modulating digestive efficiency, immune defense, and overall colony resilience during overwintering [88], emphasizing the need to assess artificial diets for their ability to support nutritional and microbial demands during overwintering and seasonal dearth.

## 5. Conclusions

This study investigated the influence of various artificial diets on the performance of *A. mellifera* colonies during dearth periods, with emphasis on diet consumption, brood development, adult bee population, enzymatic activity, worker longevity, and honey production. Among the tested formulations, diet-7 (20 g lupin flour + 20 g mung bean flour + 20 g chickpea flour + 2 g fenugreek powder + 10 g yeast + 40 g powder sugar + 10 g dry apricot powder + 10 mL vegetable oil) proved the most efficacious. It enhanced feed intake, colony development, and digestive efficiency, as indicated by elevated enzyme activity. Extended worker longevity further supported colony stability and resilience under foraging scarcity, highlighting the importance of targeted nutritional supplementation for maintaining honey bee health during nutritional stress. Future research should prioritize the optimization of artificial diets using low-cost, locally available, palatable, and shelf-stable ingredients. However, long-term, multi-seasonal evaluations are recommended to assess their effects on honey yield, queen performance, gut microbiome, and overwintering success. Additionally, investigations should examine how these diets interact with seasonal changes, particularly during overwintering, to determine whether tailored diets can enhance gut health, immunity, and colony survival under cold stress.

## Figures and Tables

**Table 1:** Composition of diet.

Diet Treatments	Diet Composition					
Diet-1	60 g lupin flour	2 g fenugreek powder	10 g yeast	40 g powder sugar	10 g dry apricot powder	10 mL vegetable oil
Diet-2	60 g mung bean flour	2 g fenugreek powder	10 g yeast	40 g powder sugar	10 g dry apricot powder	10 mL vegetable oil
Diet-3	60 g chickpea flour	2 g fenugreek powder	10 g yeast	40 g powder sugar	10 g dry apricot powder	10 mL vegetable oil
Diet-4	30 g lupin flour + 30 g mung bean flour	2 g fenugreek powder	10 g yeast	40 g powder sugar	10 g dry apricot powder	10 mL vegetable oil
Diet-5	30 g mung bean flour + 30 g chickpea flour	2 g fenugreek powder	10 g yeast	40 g powder sugar	10 g dry apricot powder	10 mL vegetable oil
Diet-6	30 g lupin flour + 30 g chickpea flour	2 g fenugreek powder	10 g yeast	40 g powder sugar	10 g dry apricot powder	10 mL vegetable oil
Diet-7	20 g lupin flour + 20 g mung bean flour + 20 g chickpea flour	2 g fenugreek powder	10 g yeast	40 g powder sugar	10 g dry apricot powder	10 mL vegetable oil
Diet-0	1 L of 50% sugar solution					

**Table 2:** Consumption of different diets in grams (g) (Mean ± SE) over a period of eight weeks.

Diets	Week 1 (g)	Week 2 (g)	Week 3 (g)	Week 4 (g)	Week 5 (g)	Week 6 (g)	Week 7 (g)	Week 8 (g)
Diet-1	37.42 ± 1.60 ^f^	36.21 ± 1.71 ^f^	35.30 ± 1.08 ^f^	36.41 ± 0.94 ^f^	43.86 ± 1.92 ^f^	39.97 ± 1.30 ^e^	43.48 ± 1.60 ^e^	43.29 ± 1.64 ^e^
Diet-2	44.66 ± 1.67 ^e^	41.83 ± 1.17 ^e^	41.20 ± 0.91 ^e^	40.6 ± 1.57 ^ef^	47.75 ± 2.21 ^ef^	42.28 ± 1.13 ^e^	46.36 ± 1.64 ^e^	46.30 ± 1.25 ^e^
Diet-3	47.76 ± 1.62 ^de^	45.46 ± 0.93 ^de^	42.86 ± 1.40 ^de^	44.63 ± 0.93 ^de^	49.84 ± 1.32 ^de^	47.74 ± 0.98 ^d^	52.25 ± 0.82 ^d^	51.59 ± 0.86 ^d^
Diet-4	51.14 ± 1.61 ^d^	47.84 ± 1.21 ^d^	46.89 ± 1.33 ^d^	48.81 ± 0.79 ^d^	54.39 ± 1.39 ^d^	50.61 ± 1.06 ^d^	55.38 ± 1.59 ^d^	54.51 ± 1.88 ^d^
Diet-5	58.22 ± 1.45 ^c^	56.12 ± 1.34 ^c^	54.15 ± 1.27 ^c^	56.12 ± 1.81 ^c^	62.51 ± 2.30 ^c^	58.76 ± 1.06 ^c^	62.32 ± 1.69 ^c^	62.00 ± 1.22 ^c^
Diet-6	65.50 ± 2.10 ^b^	62.96 ± 1.56 ^b^	62.49 ± 1.55 ^b^	63.59 ± 1.60 ^b^	70.12 ± 0.92 ^b^	65.45 ± 0.95 ^b^	70.82 ± 1.81 ^b^	70.40 ± 0.80 ^b^
Diet-7	79.90 ± 2.19 ^a^	78.37 ± 2.01 ^a^	77.58 ± 1.74 ^a^	76.04 ± 1.94 ^a^	83.41 ± 2.07 ^a^	78.73 ± 1.48 ^a^	84.29 ± 1.61 ^a^	83.30 ± 2.27 ^a^
F-value	65.2	96.8	116	94.3	61.5	145	84.70	89.5
*p*-value	<0.05	<0.05	<0.05	<0.05	<0.05	<0.05	<0.05	<0.05

Different lowercase letters within a column indicate significant differences among treatments at the 1% level (*p* < 0.05).

**Table 3:** Pearson’s correlation matrix of colony performance parameters of *Apis mellifera* fed with different artificial diets.

Variables	Brood Area	Adult Bee Population	Honey Production
Adult bee population	r = 0.856 (*p* < 0.001)		
Honey production	r = 0.856 (*p* < 0.001)	r = 0.957 (*p* < 0.001)	
Worker bee longevity	r = 0.804 (*p* < 0.001)	r = 0.920 (*p* < 0.001)	r = 0.975 (*p* < 0.001)

**Table 4:** Effect of different artificial diets on enzyme activities (amylase, lipase, proteinase, and α-glucosidase) U/mg, in honey bees (mean ± SE).

Diets	Amylase (U/mg)	Lipase (U/mg)	Proteinase (U/mg)	α-Glucosidase (U/mg)
Diet-1	46.09 ± 0.46 ^bc^	13.04 ± 0.28 ^c^	20.20 ± 0.17 ^e^	35.50 ± 0.17 ^d^
Diet-2	45.28 ± 0.43 ^c^	14.00 ± 0.29 ^c^	21.00 ± 0.14 ^d^	36.31 ± 0.18 ^d^
Diet-3	42.01 ± 0.29 ^d^	13.50 ± 0.29 ^c^	19.53 ± 0.26 ^e^	31.26 ± 0.21 ^e^
Diet-4	46.11 ± 0.35 ^bc^	15.50 ± 0.14 ^b^	22.31 ± 0.18 ^c^	37.51 ± 0.29 ^c^
Diet-5	47.50 ± 0.29 ^ab^	16.00 ± 0.14 ^b^	24.11 ± 0.12 ^b^	38.76 ± 0.15 ^ab^
Diet-6	47.00 ± 0.52 ^ab^	16.25 ± 0.14 ^ab^	23.10 ± 0.17 ^c^	38.21 ± 0.12 ^bc^
Diet-7	48.62 ± 0.23 ^a^	16.85 ± 0.20 ^a^	25.21 ± 0.18 ^a^	39.21 ± 0.21 ^a^
Diet-0	40.66 ± 0.61 ^d^	11.93 ± 0.35 ^d^	17.60 ± 0.26 ^f^	29.26 ± 0.21 ^f^
F-value	43.2	52.5	171	333
*p*-value	<0.01	<0.01	<0.01	<0.01

Different lowercase letters within a column indicate significant differences among treatments at the 1% level (*p* < 0.01).

## Data Availability

The original contributions presented in this study are included in the article. Further inquiries can be directed to the corresponding authors.
